# Advances in immunotherapy for melanoma

**DOI:** 10.1186/s12916-016-0571-0

**Published:** 2016-02-06

**Authors:** Jason M. Redman, Geoffrey T. Gibney, Michael B. Atkins

**Affiliations:** Georgetown Lombardi Comprehensive Cancer Center 3970 Reservoir Road, NW Research Building, Room E501, 20007 Washington DC, USA; Department of Medicine, Georgetown University Medical Center, Washington DC, USA

**Keywords:** Anti-PD-1, Immunotherapy, Ipilimumab, Melanoma, Nivolumab, Pembrolizumab

## Abstract

In recent years, the introduction and Federal Drug Administration approval of immune checkpoint inhibitor antibodies has dramatically improved the clinical outcomes for patients with advanced melanoma. These antagonist monoclonal antibodies are capable of unleashing dormant or exhausted antitumor immunity, which has led to durable complete and partial responses in a large number of patients. Ipilimumab targets the cytotoxic T lymphocyte-associated protein 4 (CTLA-4) receptor. Nivolumab and pembrolizumab target programmed cell death protein 1 (PD-1) receptors and have proven to be superior to ipilimumab alone. The combination of ipilimumab and nivolumab has yielded higher response rates, greater tumor shrinkage, and longer progression-free survival than either monotherapy alone. As other promising immunotherapies for melanoma proceed through clinical trials, future goals include defining the role of immune checkpoint inhibitors as adjuvant therapy, identifying optimal combination strategies, and developing reliable predictive biomarkers to guide treatment selection for individual patients.

## Background

Advanced melanoma has historically been associated with a poor prognosis, with a median overall survival (OS) of 8–10 months and a 5-year survival rate of 10 % [1]. Chemotherapy clinical trials produced modest benefit to patients, with short-lived objective responses typically seen in less than 15 % of patients [2]. Initial studies in the 1980s demonstrated the ability of interleukin-2 (IL-2) to mediate tumor regression in melanoma and other malignancies [3]. In addition, it was recognized that patients with melanoma tumors infiltrated with T cells had better long-term survival, potentially as a result of an active antitumor response by the immune system, which led to therapeutic approaches using recombinant high-dose IL-2 to induce immune-mediated tumor cell lysis in patients with metastatic melanoma [4, 5]. Pooled data from patients treated at the National Cancer Institute and within the Extramural IL-2 Working Group demonstrated objective responses in 16 % of patients treated with high-dose IL-2 [6], of which, nearly half were durable or permanent, suggesting that long-term survival or ‘cure’ is possible. However, IL-2 is associated with a number of serious toxicities, largely related to vascular leak syndrome, requiring inpatient management at experienced centers. While these factors have limited its generalized use, high-dose IL-2 serves as proof of principle that immunotherapies can eliminate tumor cells in some patients, encouraging efforts to develop better tolerated and more effective immunotherapy regimens.

In order to achieve antitumor effects, cytotoxic T lymphocytes (CTL) must not only migrate to the tumor, but must also be capable of tumor cell lysis. While the presence of tumor infiltrating lymphocytes (TIL) is frequently seen in melanoma tumors, TILs often have a diminished capacity for proliferation, cytokine production, and tumor lysis [7]. However, when TILs are removed from the tumor microenvironment (TME) and grown ex vivo, they can exhibit potent and specific antitumor activity, implying that the immune climate within the TME can dampen CTL activity. Evidence suggests that inflammation caused by immune infiltration can induce immune escape mechanisms, including interferon (IFN)-gamma-mediated upregulation of programmed death-ligand 1 (PD-L1) in the TME and increased numbers of regulatory T cells (Tregs) [8]. The engagement of PD-L1 (and PD-L2) with the programmed cell death protein 1 (PD-1) receptor on CTL leads to T cell exhaustion. Antibodies to PD-1 or PD-L1 have been shown to block the interaction between these molecules and restore antitumor immunity within the TME [9, 10].

Another mechanism of dampened immune response that is thought to predominately exert its effects in secondary lymphoid organs, as opposed to within the TME, involves cytotoxic T lymphocyte-associated protein 4 (CTLA-4) expression on T cells. CTLA-4 is a receptor exclusively expressed on T cells that binds to CD80 (B7.1) and CD86 (B7.2) on antigen-presenting cells [11]. T cell inhibition via this receptor occurs through multiple mechanisms. By outcompeting CD28 for binding to B7.1 and B7.2, CTLA-4 can prevent the co-stimulation necessary to generate and maintain T cell activation. Additionally, evidence suggests that expression of CTLA-4 on Tregs is important in T cell inhibition [12]. Antibodies to CTLA-4 have been shown to block the interaction between CTLA-4 and its ligands, restoring the function of T cells in the antigen-presenting compartment [13].

Clinical development of monoclonal antibodies that block CTLA-4 and PD-1 have been major advances in cancer immunotherapy. Ipilimumab, an antagonist monoclonal antibody (mAb) to CTLA-4, was first approved by the Federal Drug Administration.

(FDA) for treatment of patients with advanced melanoma in 2011. Pembrolizumab and nivolumab are both antagonist mAbs to PD-1 and were FDA approved in 2014. The ability of these checkpoint inhibitors to induce durable complete and partial tumor responses has ushered in a new era for the treatment of patients with advanced melanoma. The high therapeutic index of pembrolizumab and nivolumab has prompted their study in the adjuvant setting in patients with resected high-risk melanoma, both in combination with each other as well as with other novel immunotherapy agents, in patients with advanced disease. Research is ongoing to identify biomarkers that can guide the selection of immunotherapy for individual patients. All of these approaches hold promise for further improvement in the outcomes of patients with melanoma.

### Anti-CTLA-4 therapy

Ipilimumab demonstrated clinical activity in early phase trials [14–16] and was approved by the FDA following the release of phase III data, which showed a significantly improved OS relative to the glycoprotein 100 (gp100) peptide vaccine in previously treated melanoma patients [17]. Median OS in patients receiving ipilimumab plus gp100 and ipilimumab alone were 10.0 and 10.1 months, respectively, versus 6.4 months in those that received gp100 alone. The more striking findings from this study were the ipilimumab 1- and 2-year OS rates for the ipilimumab-alone arm, of 45.6 % and 23.5 %, respectively, as well as similar rates for the ipilimumab plus gp100 arm. The 1-year OS rate was higher than had previously been reported using any other experimental regimen for patients with untreated advanced melanoma. In a second phase III trial, where advanced melanoma patients were randomized to ipilimumab plus dacarbazine versus dacarbazine alone, median OS was superior in those who received ipilimumab (hazard ratio (HR) for death was 0.72, *P* <0.001) [18]. However, the combination of ipilimumab plus dacarbazine has not been accepted as a standard approach due to the increased risk of hepatotoxicity coupled with only a relatively modest increase in clinical activity over ipilimumab alone.

Pooled data from 10 prospective and two retrospective studies on ipilimumab-treated patients with advanced melanoma confirmed that long-term survival is possible [19]. The Kaplan–Meier survival curve of treated patients reached a plateau at 3 years with 22 % of patients alive. Follow-up was extended to 10 years and it was suggested that durable OS with ipilimumab could be achieved. Subset analyses showed slightly better survival in patients who were treatment naive, but no substantial difference in survival was observed for patients treated with ipilimumab at 3 mg/kg compared to 10 mg/kg dose levels. The question of a difference in efficacy based on dose level is currently being tested in a randomized phase III trial of ipilimumab 3 mg/kg versus 10 mg/kg in patients with metastatic melanoma (NCT01515189).

Tremelimumab, another mAb targeting CTLA-4, displayed activity in a phase II study with an objective response rate (ORR) of 9.8 % and 9.3 % in groups receiving 10 mg/kg every month and 15 mg/kg every 3 months, respectively [20]; the respective 12-month OS rates were 32 % and 46 %. However, a randomized phase III study of tremelimumab versus chemotherapy failed to demonstrate a survival advantage [21]; nevertheless, data from this open-label study may have been affected by crossover of patients in the chemotherapy arm to ipilimumab, possibly confounding any potential survival difference. Evaluation of tremelimumab’s activity in combination with other agents is ongoing (discussed below).

While ipilimumab increases immune activity against tumor cells, it can also break immune tolerance to self and cause autoimmune side effects. Such immune-related adverse events (irAE) most commonly manifest as dermatitis, colitis, hepatitis, hypophysitis, and thyroiditis [17]. A meta-analysis (in subjects with various malignancies including melanoma) calculated an overall incidence of irAEs in 72 % of ipilimumab-treated patients, with a 24 % incidence of high-grade adverse events [22]. Fortunately, irAEs are responsive to corticosteroid therapy or other immune suppressive agents and tumor responses can occur even after treatment is stopped to initiate immunomodulatory therapy [17, 23]. Further, distinctive to checkpoint inhibitor therapies, approximately 10 % of patients who receive ipilimumab will initially experience ‘pseudoprogression’, wherein tumors appear to grow larger or new lesions develop, likely due to enhanced immune effector cell infiltration, and only subsequently exhibit tumor shrinkage. These adverse events and response characteristics led to the development of the irAE toxicity designation and immune-related response criteria for adequate characterization of the effects of ipilimumab treatment [24].

### Anti-PD-1/PD-L1 therapy

Soon after the development of ipilimumab, data describing the clinical activity of the anti-PD-1 mAb nivolumab in patients with advanced malignancies emerged [25, 26]. In patients with advanced melanoma, non-small cell lung cancer (NSCLC), and renal cell cancer, objective responses were seen in 17–34 % of patients with median response durations of 13–24 months. Nivolumab also appeared to have a favorable adverse event profile, with treatment-related grade 3–4 toxicities typically occurring in less than 15 % of patients [26, 27]. OS rates for patients with melanoma were 62 % at 1 year, 43 % at 2 years, and 41 % at 3 years [27, 28]. The phase I trial of the anti-PD-1 mAb pembrolizumab (KEYNOTE-001) also showed strong clinical activity [29]. Pembrolizumab produced durable responses in both ipilimumab-naive and previously treated patients with melanoma with an ORR of 33 % [30]. Median duration of response had not yet been reached, with a majority of patients continuing on active therapy.

Subsequent trials confirmed the efficacy of both nivolumab and pembrolizumab in patients with advanced melanoma. Weber et al. [31] reported on the randomized phase III trial of nivolumab versus investigator’s choice chemotherapy in patients with melanoma whose disease had progressed after ipilimumab and a BRAF inhibitor if the tumor contained a BRAF V600 mutation (Checkmate-037). The study met its primary endpoint of superior ORR in the nivolumab group, which was 31.7 %, compared to an ORR of 10.6 % with chemotherapy. At the time of the analysis, 87 % of responses were ongoing. The co-primary endpoint of improved OS has not yet been reported. In the randomized phase II trial of pembrolizumab compared to physician’s choice of chemotherapy in a similar patient population, superior clinical activity was also shown with pembrolizumab (KEYNOTE-002) [32]. The ORRs were 25 % and 21 % for the 10 mg/kg and 2 mg/kg dose levels of pembrolizumab, respectively, and 4 % for chemotherapy. Median progression-free survival (PFS) was 5.6 and 5.4 months for the pembrolizumab arms versus 3.6 months for the chemotherapy arm. Crossover from chemotherapy to pembrolizumab was permitted, confounding the OS assessments.

IrAEs and other treatment-related adverse events can be seen with anti-PD-1 therapies, although rates of severe events (grade 3–5) have been lower than those seen with ipilimumab, ranging from 8–16 % of patients treated with either pembrolizumab or nivolumab [19, 26, 33]. The most common reported treatment-related adverse events have been fatigue, pruritus, rash, arthralgia, nausea, diarrhea, and hypothyroidism. Severe cases of colitis, dermatitis, pneumonitis, and hepatitis have been typically reported in 1 % or less of patients. Severe irAEs can be managed by holding or discontinuing the anti-PD-1 agent and administering high-dose corticosteroids followed by other immune modulatory agents if side effects are not quickly controlled.

Experience with anti-PD-L1 antibodies as monotherapy in patients with advanced melanoma has been limited. One out of eight melanoma patients on the phase I trial of durvalumab (MEDI4736) achieved a partial response [34]. Data from the phase I trial of atezolizumab (MPDL3280A) in locally advanced or metastatic melanoma patients showed an ORR of 26 % as well as several patients with delayed antitumor activity that was not included in the ORR [35]. Grade 3–4 adverse events (regardless of attribution) were seen in 33 % of patients, which included hyperglycemia (7 %) and transaminitis (7 %). No cases of grade 3–5 pneumonitis were observed.

#### Anti-PD-1 therapy versus ipilimumab

Anti-PD1 therapy has now been compared head-to-head with ipilimumab in the first line setting in patients with metastatic melanoma. KEYNOTE-006 was a phase III trial comparing standard ipilimumab to pembrolizumab at 10 mg/kg every 2 or every 3 weeks in patients with melanoma who were naive to checkpoint inhibitor therapy [36]. ORR was similar for both pembrolizumab schedules (33.7 % for every 2 weeks and 32.9 % for every 3 weeks) but clearly higher than the ORR with ipilimumab (11.9 %); the corresponding 6-month PFS rates were 47.3 %, 46.4 %, and 26.5 %, respectively. Further, 1-year OS was higher with pembrolizumab (64.8–74.1 % vs. 58.2 % for ipilimumab) and the emergence of severe treatment-related adverse events was lower in patients receiving pembrolizumab compared to ipilimumab (10.1–13.3 % vs. 19.9 %).

The Checkmate-067 study was a randomized phase III trial of ipilimumab monotherapy compared to nivolumab monotherapy or the combination of nivolumab and ipilimumab in patients with advanced melanoma who were naive to immune checkpoint inhibitor therapy [37]; the ORR was 43.7 % with nivolumab compared to 19.0 % with ipilimumab. A longer PFS (HR, 0.57; *P* <0.001; co-primary endpoint) and lower toxicity were seen with nivolumab monotherapy as well. Data on OS has not yet been reported. Thus, the data from KEYNOTE-006 and Checkmate-067 confirm the clinical superiority of anti-PD-1 therapy over anti-CTLA-4 therapy in patients with advanced melanoma.

### Immune checkpoint blockade for melanoma brain metastases (MBM)

Initial data from the phase III trial of ipilimumab with or without the gp100 vaccine and an ipilimumab expanded access program suggested clinical activity in a subset of patients with MBM without additional toxicities [17, 38]. This led to the phase II study of ipilimumab in patients with melanoma with previously untreated brain metastases, which included a cohort of asymptomatic, non-steroid-dependent patients and a cohort of symptomatic patients requiring corticosteroids [39]. In the first cohort, ipilimumab led to an intracranial ORR of 16 % and an intracranial disease control rate of 25 %. While median OS was short (7.0 months), 24 % of patients were alive at 2 years, indicating long-term survival may also be possible in a subset of patients with MBM treated with ipilimumab. In the cohort of patients with symptomatic MBM requiring steroids, the intracranial ORR and disease control rates were notably lower (5 % and 10 %, respectively), as were the median OS (3.7 months) and 2-year OS (10 %) rates. Similar irAEs were seen in this MBM study compared to other ipilimumab studies. The most common events were diarrhea, rash, pruritus, and elevated serum transaminase levels. Infrequent headaches, dizziness, brain hemorrhage, and brain edema were also reported; however, the low incidence suggests most were probably related to the central nervous system (CNS) disease rather than increased toxicity from ipilimumab.

As with ipilimumab, anti-PD-1 studies permitted enrollment of melanoma patients with treated brain metastases, but this has generally represented less than 10 % of the total accrued population. Preclinical data has suggested a potential role for the treatment of active MBM with anti-PD-1 therapy [40]. However, it is not clear if CNS penetration of the monoclonal antibodies is possible or required to generate antitumor immune responses with this class of therapy. Early data from an ongoing phase II study of pembrolizumab in patients with active MBM was reported at the ASCO 2015 General Meeting [41]. Out of 12 evaluable patients, three patients had intracranial partial response (one of these subjects had received prior ipilimumab). Two additional patients had stable intracranial disease. The three partial responses were durable for 7+, 6+, and 3+ months at the time of the report. There were no significant treatment-related CNS adverse events noted.

### Adjuvant therapy for resected melanoma

Five-year survival rates in patients with resected stage III melanoma have ranged from 70 % in patients with stage IIIA disease to as low as 39 % in patients with stage IIIC disease [42]. The role for adjuvant systemic therapy in this setting and in cases of completely resected stage IV melanoma has been studied in numerous trials. Both high-dose IFN-alpha-2b and pegylated IFN-alpha-2b have demonstrated improved relapse-free or disease-free survival in randomized clinical trials and are approved by the FDA for use in this setting [43, 44]. However, improvement in OS has been inconsistent across trials [45]. More recently, biochemotherapy was shown to yield significantly longer relapse-free survival compared to high-dose IFN in a randomized phase III clinical trial conducted by the Southwest Oncology Group (S0008) [46]; however, no difference was seen in OS and biochemotherapy was associated with a higher severe toxicity rate. The clinical activity and tolerability of checkpoint inhibitors in patients with advanced melanoma provides a rationale for investigation in the adjuvant setting.

Ipilimumab has now been studied in two randomized phase III trials compared to placebo (EORTC 18071) or high-dose IFN (ECOG 1609). In the EORTC 18071 trial, patients with resected stage III cutaneous melanomas were randomized to ipilimumab 10 mg/kg or placebo every 3 weeks for four doses, then every 3 months for up to 3 years [47]. Results showed improved median recurrence-free survival of 26.1 months with ipilimumab compared to 17.1 months with placebo (HR, 0.75; *P* = 0.0013). In subgroup analyses, patients with microscopic lymph node disease or ulcerated primary lesions demonstrated the most benefit. Also important to note was the high rate of grade 3–5 irAEs seen in patients receiving ipilimumab in this study (43 % vs. 2 % with placebo). These included five treatment-related deaths (colitis n = 3, myocarditis n = 1, and multiorgan failure with Guillan–Barre syndrome n = 1), despite management with immunomodulatory therapy. OS data is not yet mature. While this data is provocative, and has led to the recent FDA approval of ipilimumab for patients with resected stage III melanoma, it is as yet unclear whether the reduction in recurrence rate with ipilimumab represents an improvement over adjuvant IFN therapy and whether this benefit will translate into an improvement in OS. The former question is being addressed by the ongoing E1609 study, which randomized patients with resected stage III–IV melanoma to ipilimumab 10 mg/kg or 3 mg/kg or high-dose IFN [48]. The study completed accrual of more than 1,500 patients in the summer of 2014 and is pending analysis for primary endpoints of relapse-free survival and OS. Long-term survival data from both of these adjuvant studies will ultimately be necessary in order to determine the true impact of adjuvant ipilimumab therapy.

PD-1 inhibitors have proven to be less toxic and more active than ipilimumab in patients with established, unresectable metastatic melanoma [36, 37]. Given the favorable therapeutic index, there is much interest in developing this class of therapies as adjuvant treatment for patients with high-risk resected melanoma. Results from a phase I trial of nivolumab plus a multi-peptide vaccine in 33 patients with resected stage IIIc or IV melanoma showed a relatively low relapse rate (30 %) during a median follow-up period of 32.1 months from trial enrollment. Median relapse-free survival was estimated to be 47.1 months [49]. Phase III trials with nivolumab and pembrolizumab in patients with resected stage III and IV melanoma are currently underway. These include Checkmate-238, comparing ipilimumab 10 mg/kg to nivolumab 3 mg/kg, which completed accrual in September of 2015; the EORTC 1352 (KEYNOTE-054) protocol, comparing pembrolizumab (200 mg flat dose) to placebo, which is actively accruing patients; and the SWOG S1404 protocol, comparing pembrolizumab (200 mg flat dose) to high-dose IFN, which is also actively accruing patients (NCT02506153).

### Anti-PD-1/PD-L1 combination immune therapy strategies

#### Anti-PD-1/PD-L1 plus anti-CTLA-4

Preclinical murine studies verified the hypothesis that, in light of their distinct mechanisms, combining CTLA-4 and PD-1 blockade could augment antitumor activity beyond that of either strategy alone. Combination therapy increased the degree of tumor response and was associated with greater numbers of effector T cells and less Tregs in the TME in murine models involving syngeneic implants of either colon cancers or melanoma [50]. A phase I trial of nivolumab plus ipilimumab in patients with advanced melanoma demonstrated an ORR of 43 % and 1- and 2-year OS rates of 85 % and 79 %, respectively [51, 52]. The rate of grade 3–4 treatment-related adverse events was substantially higher (>60 %) compared to the rates previously seen with anti-CTLA-4 or anti-PD-1 monotherapy. However, these events were similar to those seen with ipilimumab monotherapy and were also manageable with early institution of high-dose corticosteroids or other immune modulatory agents.

Subsequently, two randomized studies (Checkmate-069 and Checkmate-067) were conducted to compare combined immunotherapy with nivolumab plus ipilimumab to immune checkpoint inhibitor monotherapy. The Checkmate-069, a double-blind phase II trial, randomized patients to ipilimumab 3 mg/kg plus nivolumab 1 mg/kg or placebo every 3 weeks, followed by nivolumab 3 mg/kg or placebo every 2 weeks until disease progression or toxicity requiring study withdrawal [53]. In patients with BRAF-wildtype tumors, the ORR was 61 % in the group that received nivolumab plus ipilimumab, compared to 11 % in the ipilimumab plus placebo group. Median PFS was 4.4 months in the ipilimumab monotherapy group, whereas median PFS was not reached for the combination group at the time of analysis. There were 16 patients (22 %) with complete responses in the combination group, and none in the ipilimumab-monotherapy group. A subset of patients with BRAF mutant tumors were observed to have similar ORR and PFS to those in the larger study, suggesting that tumor BRAF status has no effect on response to checkpoint inhibitor therapy. This favorable data clearly established that combination therapy produced superior antitumor efficacy to ipilimumab in patients with BRAF-wildtype melanoma and led to the FDA approval of the combination for this patient population in October 2015.

As mentioned earlier, the Checkmate-067 trial was a three-arm, double blind, phase III trial that randomized patients to nivolumab 3 mg/kg every 2 weeks or nivolumab 1 mg/kg every 3 weeks plus ipilimumab 3 mg/kg every 3 weeks for four doses, followed by nivolumab 3 mg/kg every 2 weeks or ipilimumab 3 mg/kg every 3 weeks for four doses [37]. While the study was not preplanned for a statistical comparison between nivolumab plus ipilimumab versus nivolumab monotherapy, the data provides insight into how these two strategies compare to each other. The ORR was 57.8 % in patients receiving the combination therapy compared to 43.7 % in patients receiving nivolumab monotherapy. Response was independent of tumor BRAF mutational status. At the time of publication, OS data had not yet matured; however, overall tumor shrinkage was greater (51.9 % vs. 34.5 %) and median PFS was longer in those patients receiving the combination compared to nivolumab monotherapy (11.5 months vs. 6.5 months; HR, 0.74; 95 % confidence interval, 0.60–0.92).

The results from the Checkmate-069 and -067 studies establish that the combination produces impressive antitumor activity and suggests that all of the antitumor effects of immunotherapy are not subsumed in the activity of single agent PD-1 blockade. However, the combination of nivolumab + ipilimumab also produces a clear increase in severe treatment-related adverse events. In Checkmate-069, the nivolumab plus ipilimumab group had a grade 3–5 adverse event rate of 54 % compared to a rate of 24 % observed in the ipilimumab-alone group [53]. In Checkmate-067, grade 3–4 adverse events were seen at a rate of 55 % in the nivolumab plus ipilimumab group, compared to 16 % in the nivolumab group and 27 % in the ipilimumab group [37]. While there were three reported deaths in the combination therapy group that were attributable to checkpoint inhibitor therapy in the phase II trial [53], there were none in the phase III trial. Similar to checkpoint inhibitor monotherapy, timely recognition of irAEs and treatment with immunomodulators can control these side effects in most patients receiving the combination. More importantly, stopping treatment does not preclude derivation of benefit from treatment. While 36 % of patients had treatment discontinuation on Checkmate-069, 67 % of these patients had an objective response that continued on past discontinuation of therapy [37, 53].

In light of the high toxicity profile of nivolumab plus ipilimumab combination despite its clinical activity, alternative combination strategies are now being explored, including a randomized phase II sequencing trial of nivolumab followed by ipilimumab versus ipilimumab followed by nivolumab in patients with advanced melanoma (Checkmate-064) [54]; the cumulative ORRs at week 25 were 47.7 % and 22.6 %, respectively, suggesting higher clinical activity in patients who receive nivolumab first. Unfortunately, the cumulative rates of grade 3–5 treatment-related adverse events remained high with both sequencing approaches (50 % and 43 %, respectively). Combination of pembrolizumab with a lower dose of ipilimumab (1 mg/kg) is also currently being studied in advanced melanoma patients enrolled in the KEYNOTE-029 trial. Preliminary data showed antitumor activity and perhaps less toxicity [55]. Dose expansion of this combination in patients with melanoma is ongoing with results pending. In a comparable approach, the anti-PD-L1 mAb durvalumab is being combined with the CTLA-4 mAb tremelimumab in a phase I trial (NCT02537418).

#### Anti-PD-1 in combination with cytokine therapy

The clinical activity of combination anti-PD-1 and anti-CTLA-4 therapies provides proof of principal that efficacy seen with anti-PD-1 monotherapy can be enhanced by the addition of other non-redundant immunotherapies. Past studies combining cytokines with ipilimumab, such as IFN-alpha-2b and granulocyte macrophage-colony stimulation factor (GM-CSF), have suggested enhanced clinical activity, which provides merit for combining such agents with PD-1 pathway blockers. A single center phase I/II study of pegylated IFN (1–3 μg/kg weekly) in combination with standard ipilimumab dosing showed an ORR of 47 % and 1-year OS of 56 % [56]. In a randomized phase II study of ipilimumab (10 mg/kg) with or without GM-CSF, the ORRs were similar (15.5 % vs. 14.8 %, respectively), but a significantly longer OS was demonstrated in the combination group (1-year OS rate of 68.9 % vs. 52.9 %, *P* = 0.01) [57]. Interestingly, fewer grade 3–5 toxicities were observed in the combination group compared to ipilimumab monotherapy (45 % vs. 58 %). Both of these studies have led to investigation of cytokines in combination with anti-PD-1-based regimens.

The combination of pegylated IFN and pembrolizumab has now been investigated in two separate clinical trials (NCT02112032 and NCT02089685). Preliminary results of the single center study were presented in abstract form at the 2015 ASCO General Meeting [58]. In this phase I trial, three dose levels of weekly pegylated IFN (1, 2, and 3 μg/kg weekly) were combined with pembrolizumab at 2 mg/kg every 3 weeks. The combination was reasonably tolerated in the first 12 patients and clinical activity was seen in the six evaluable patients. The second trial is KEYNOTE-029, where it was studied in patients with advanced melanoma and renal cell carcinoma; data from this study has not been published. With regards to GM-CSF, a randomized phase III intergroup trial of nivolumab plus ipilimumab with or without sargramostim (EA6141) is currently recruiting subjects with advanced melanoma and should provide more guidance (NCT02339571).

#### Anti-PD-1/PD-L1 in combination with novel immune agents

Talimogene laherparepvec (T-VEC) is an oncolytic virus (modified herpes simplex virus) that expresses GM-CSF, which is injected directly into the tumor to generate an antitumor immune response. A randomized phase III study (OPTiM trial) in patients with unresectable stage IIIb–IV melanoma comparing intralesional T-VEC therapy to subcutaneous GM-CSF therapy demonstrated an overall durable response rate of 16.3 % (2.1 % for GM-CSF arm) [59]. This included tumor regression in injected tumor sites as well as occasional regression in non-injected tumor sites. While OS was not significantly improved, there appeared to be a strong trend toward greater benefit in those patients receiving T-VEC relative to GM-CSF alone, particularly in those with regional disease only. The application of T-VEC therapy for local immune stimulation in a combination immune checkpoint blockade could provide enhanced clinical activity. The combination of T-VEC with ipilimumab is being studied in an ongoing phase Ib/II trial of patients with metastatic melanoma and at least one injectable lesion [60]. Early data has demonstrated an ORR of 56 % (33 % complete response rate) with a median PFS of 10.6 months. OS at 12 and 18 months was 72.2 % and 67 %, respectively; however, these results are likely influenced by the inclusion of stage III patients in the study. T-VEC is also being studied in combination with anti-PD-1 therapy. A randomized, open label trial of T-VEC plus pembrolizumab versus pembrolizumab alone is actively enrolling patients with unresectable stage IIIB–IV melanoma and at least one injectable lesion (NCT02263508).

Another promising immunotherapy target for combination strategies is indoleamine 2,3-dioxygenase 1 (IDO_1_), which is upregulated in malignant cells and myeloid-derived suppressor cells and converts tryptophan to kynurenine, leading to immune suppression in the TME [61, 62]. While monotherapy with IDO_1_ inhibition has not demonstrated robust clinical activity [63], promising results have been demonstrated with the combination of the IDO_1_ inhibitor epacadostat (INCB024360) and ipilimumab in patients with advanced melanoma. From the phase I trial, a dose of epacadostat up to 50 mg twice daily in combination with standard ipilimumab was generally well tolerated and with an ORR of 31 % (10 out of 32 immunotherapy-naive patients) [64]. In vivo studies have also demonstrated a synergistic effect when combining IDO_1_ inhibition with PD-1 blockade [65]. A phase I/II trial of pembrolizumab plus epacadostat in multiple malignancies including melanoma is currently underway. Preliminary data presented at the 2015 SITC general meeting showed objective responses in four out of seven patients with melanoma evaluable at the time of the report. Across all malignancies in this study, there were few grade 3 adverse events and no grade 4 events [66]. In addition, clinical trials with epacadostat in combination with other anti-PD-1/PD-L1 therapies (including nivolumab, durvalumab, and atezolizumab) are currently enrolling patients.

Multiple novel immune checkpoint agonists and antagonists as monotherapy and in combination are in development, including stimulatory mAbs directed at 4-1BB, OX40, CD27, and GITR on T cells in the TME. Of these targets, several are already planned for combination phase I/II studies with PD-1 pathway inhibitors, such as the 4-1BB agonist PF-05082566 plus pembrolizumab (NCT02179918), the OX40 ligand fusion protein MEDI6383 in combination with durvalumab (NCT02221960), and the CD27 agonist varililumab in combination with nivolumab (NCT02335918). Blockade of immune suppressive targets, such as LAG-3 and TIM-3, may also hold promise alone or in combination with PD-1 pathway inhibitors. Indeed, preclinical data have shown that in vivo co-inhibition or knock-out of LAG-3 and PD-1 demonstrated robust immune activation, tumor rejection, and abrogation of self-tolerance [67, 68]. Further implying a role for anti-LAG-3 and anti-PD-1 combination therapy, a recent study on banked melanoma tumor samples showed the LAG-3 gene to be overexpressed in PD-L1 positive tumors [69]. The anti-LAG-3 mAb BMS-986016 is currently being studied in a phase I trial as monotherapy and in combination with nivolumab in patients with advanced solid tumors (NCT01968109). These new checkpoint agents may eventually prove to be effective alternatives to ipilimumab for combination with the anti-PD-1 blockade as upfront therapy or after progression with anti-PD-1/PD-L1 monotherapy.

### Biomarkers

The development of predictive biomarkers for immunotherapies in melanoma has been an area of great research interest. Past studies examining biomarkers associated with clinical benefit from high-dose IL-2 have yielded several potential strategies, including circulating vascular endothelial growth factor and fibronectin levels or T cell gene expression patterns on tumor biopsies [70, 71]. However, these have not been validated in prospective trials. In this era of checkpoint inhibitor therapy, identification of a population that benefits as much from anti-PD-1 monotherapy as combination anti-PD-1 plus ipilimumab would be useful as it could spare patients the increased risk of severe adverse events from combination therapy. Research into predictive biomarkers for anti-PD-1-based therapies has largely focused on PD-L1 expression, but other promising strategies are now emerging.

#### PD-L1 expression

Data from the phase I study of nivolumab suggested a potential role for use of tumor PD-L1 immunohistochemistry (IHC) as a predictive biomarker for anti-PD-1 therapy [26]. Nine of 25 patients (36 %) with PD-L1-positive disease demonstrated an objective response to nivolumab, whereas none of the 17 PD-L1-negative tumor patients had an objective response. Subsequent studies have demonstrated higher response rates with anti-PD-1 therapies in patients whose tumors are PD-L1 positive [72]. However, objective responses in patients with PD-L1 negative tumors have been observed in most studies with ORRs ranging from 11–20 % and as high as 41.3 % in Checkmate-067 [37]. Therefore, refraining from the use of anti-PD-1/PD-L1 agents in patients whose tumors test negative for PD-L1 status would potentially prevent access to an effective therapeutic strategy in a large number of patients. Furthermore, the use of PD-L1 as a predictive biomarker is complicated by the lack of uniformity in the antibody used for PD-L1 detection and thresholds for cutoff of PD-L1 positive and negative status across studies [73]. For example, the PD-L1 assay developed as a biomarker for pembrolizumab studies uses an antibody against the 22C3 region of PD-L1 and a ‘proportional score’ of ≥1 % (melanoma) for PD-L1-positive disease, which has been observed in 80 % of melanoma tumors [32, 36]. A similar PD-L1 assay for nivolumab uses an antibody targeting the 28-8 region of PD-L1 for IHC and uses a cutoff of 5 % (1 % and 10 % cutoff points have also been studied), where 24–50 % of melanoma tumors test positive [31, 37].

Despite these limitations, the use of PD-L1 IHC is important for stratification of patients on anti-PD-1/PD-L1 therapy trials. It may also play a role in the selection of immunotherapy strategies in patients with melanoma and other malignancies. Data on patients with advanced NSCLC from the phase I study of pembrolizumab (KEYNOTE-001) and the phase III study of nivolumab (non-squamous only; Checkmate-057) is probably the strongest so far for clinical application of PD-L1 testing. From the KEYNOTE-001 study, the ORR was enriched 3- to 4-fold and OS was not reached after a median follow-up of 10.9 months in NSCLC patients with a PD-L1 expression proportional score of ≥50 % [74]. Similarly, in Checkmate-057, PD-L1-positive patients (5 % cutoff) showed significant improvement in OS with nivolumab over docetaxel (HR, 0.43; *P* <0.001), which was not observed in PD-L1-negative patients [75]. With regards to melanoma, the Checkmate-067 study demonstrated similar PFS between nivolumab monotherapy and nivolumab plus ipilimumab combination arms (median PFS was 14 months in both) in PD-L1-positive patients [37]. However, the ORR was greater with the nivolumab plus ipilimumab combination therapy compared to nivolumab monotherapy (72.1 % vs. 57.5 %, respectively) in PD-L1-positive patients and OS data is not yet mature. As such, the value of tumor PD-L1 expression in choosing combination versus monotherapy remains to be determined.

In order for PD-L1 status to move forward as an effective predictive biomarker, PD-L1 assays will likely need to be standardized and associations confirmed in prospective studies. Because of the intra-tumor and patient heterogeneity of PD-L1 status and the inducible nature of PD-L1 [73], additional biomarker approaches will likely be needed to adequately predict likelihood of response to checkpoint inhibitors.

#### Emerging biomarker strategies associated with anti-PD-1/PD-L1 therapy

Tumeh et al. [76] demonstrated that response to anti-PD-1 therapy appears to rely on a pre-treatment presence of PD-1- and PD-L1-expressing cells at a close interface, as well as the presence of CD8+ TILs. This study describes what are likely the components of phenotypic patterns of immune interaction governing the immune resistance of tumors along this spectrum. The activity of TILs is included in these analyses, as it has been shown that their secretion of IFN-gamma can induce PD-L1 expression in tumor cells [77, 78]. Analysis of gene expression in responders to pembrolizumab from the KEYNOTE-001 study revealed an increase in the expression of IFN-gamma-associated genes [79].

Tumeh et al. [76] identified the presence of CD8+ TIL in patients who responded to pembrolizumab and hypothesized that this infiltrate would have a narrowed repertoire of T cell receptors that enable a tumor-specific immune response. Next generation sequencing of pre-treatment tumor samples revealed a less diverse and more clonal population of T cells [76]. Moreover, post-treatment, biopsies revealed 10-fold greater T cell receptor clonal expansion when compared to pre-treatment biopsies. As proposed by the authors, these data indicate that PD-1/PD-L1 expression may be an indirect marker of activated CD8+ TIL within the TME. It is this activity that may be driving adaptive immune escape by tumors via the PD-1/PD-L1 axis and other mechanisms. Further, this presence seems to correlate with response to anti-PD-1 therapy. These findings provide a basis from which to hypothesize that addition of ipilimumab to anti-PD-1 inhibitor therapy can provide additional immune support in patients without brisk CD8+ TIL infiltration.

Other works offer mutational burden and the presence of neoantigens as a potential marker of response to anti-CTLA-4 and anti-PD-1 therapies. A study of 64 melanoma patients treated with ipilimumab or tremelimumab analyzed the association of mutational load based on tumor whole exome sequencing and clinical benefit (disease control for at least 6 months) [80]. A significant correlation between mutational load (>100 non-synonymous somatic mutations) and clinical benefit was seen. Furthermore, derivation of a neoepitope signature for major histocompatibility complex class I presentation from this data was highly associated with clinical outcome, providing a strong scientific explanation for this observation. A similar study was recently published [81], where tumor samples of 110 melanoma patients treated with ipilimumab were analyzed via whole exome sequencing. These data also demonstrated that mutational and neoantigen loads were associated with clinical benefit from ipilimumab. However, identified neoantigens rarely recurred among patients. As the authors suggest [78], larger cohorts will likely be required to identify markers predictive of clinical benefit with checkpoint inhibitor therapy in future studies. Interestingly, a large-scale genetic study on banked tumor samples of many different malignancies analyzed genes of TIL and tumor cells [82], revealing neoantigen presence as a strong predictor of cytolytic activity and highlighting several mutations associated with less cytolytic activity than expected. Another analysis of mutational burden has been associated with clinical outcomes in patients with NSCLC treated with pembrolizumab [83].

Further investigation into the relationship of PD-1/PD-L1 expression, TIL presence, T cell repertoire, and mutational burden should be aimed at creating a model by which response to anti-PD-1/PD-L1-based therapies can be predicted. In such a model, different profiles may help select patients who will have optimal benefit with anti-PD-1/PD-L1 monotherapy and/or direct towards various combination approaches.

## Conclusions

The introduction of checkpoint inhibitor immunotherapies has ushered in a new era in the treatment of patients with melanoma. Anti-CTLA-4- and anti-PD-1-based approaches have expanded upon the successes seen with systemic IL-2 and can produce response rates above 50 % when administered in combination. While the efficacy of these new therapies is enhanced, the toxicity is less severe than that seen with high-dose IL-2. The toxicities from checkpoint immunotherapy represent a new class of adverse events, termed irAEs, manageable with early application of systemic corticosteroids or immunomodulators and possible predictors of favorable PFS and OS [84]. Remarkably, immunosuppressive therapy does not appear to dampen ongoing antitumor effects [85].

In evaluating response to these new therapies, there appears to be a spectrum of patients ranging from those in which blocking the PD-1/PD-L1 axis alone is effective to those who respond better with the addition of CTLA-4 blockade and, finally, to those who do not respond to either strategy. Novel immunotherapies are in the clinical pipeline and will hopefully provide effective options for those who do not respond to anti-PD-1-based combination approaches.

While the OS data from the Checkmate-067 study are not yet mature, it is clear that the combination of anti-CTLA-4 and anti-PD-1 therapy produces a better ORR and median PFS, but also greater toxicity, than either monotherapy. Therefore, a predictive model based on multiple biomarkers will likely be needed to select patients who will require combination treatment regimens with higher toxicity rates in order to maximize antitumor responses. Despite early data identifying expression of PD-L1 on tumor cells as being associated with response to anti-PD-1/PD-L1 monotherapy, that characteristic alone is not currently suitable for clinical decision-making in patients with melanoma. While standardization of PD-L1 assays will be useful, multiple biomarkers beyond PD-L1 status will likely need to be incorporated in order to achieve the precision required for guiding therapeutic choices in individual patients. Likely candidates include CD8+ T cell density and geographic associations with PD-L1, IFN-gamma gene expression signatures, T cell clonality, and mutational burden/neo-epitope signatures. Sampling patient tumors in the pre-treatment setting for tumor immune phenotypes or composite biomarker profiles is likely to become a standard process in immunotherapy planning for patients with melanoma and other immune responsive tumors.

## Abbreviations

CNS: Central nervous system
CTL: Cytotoxic T lymphocyte
CTLA-4: Cytotoxic T lymphocyte-associated protein 4
FDA: Federal Drug Administration
GM-CSF: Granulocyte macrophage-colony stimulation factor
gp100: Glycoprotein 100
IDO_1_: Indoleamine 2,3-dioxygenase 1
IFN: Interferon
IHC: Immunohistochemistry
IL-2: Interleukin-2
irAE: Immune-related adverse event
mAb: Monoclonal antibody
MBM: Melanoma brain metastases
NSCLC: Non-small cell lung cancer
ORR: Objective response rate
OS: Overall survival
PD-1: Programmed cell death protein 1
PD-L1: Programmed death-ligand 1
PFS: Progression-free survival
TIL: Tumor infiltrating lymphocyte
TME: Tumor microenvironment
Tregs: Regulatory T cells
T-VEC: Talimogene laherparepvec

## References

[1] Garbe C, Eigentler TK, Keilholz U, Hauschild A, Kirkwood JM (2011). Systematic review of medical treatment in melanoma: current status and future prospects. Oncologist.

[2] Middleton MR, Grob JJ, Aaronson N, Fierlbeck G, Tilgen W, Seiter S (2000). Randomized phase III study of temozolomide versus dacarbazine in the treatment of patients with advanced metastatic malignant melanoma. J Clin Oncol.

[3] Rosenberg SA, Lotze MT, Muul LM, Leitman S, Chang AE, Ettinghausen SE (1985). Observations on the systemic administration of autologous lymphokine-activated killer cells and recombinant interleukin-2 to patients with metastatic cancer. N Engl J Med.

[4] Pastorfide GC, Kibbi AG, de Roa AL, Barnhill RL, Sober AJ, Mihm MC (1992). Image analysis of stage 1 melanoma (1.00-2.50 mm): lymphocytic infiltrates related to metastasis and survival. J Cutan Pathol.

[5] Sober AJ, Day CL, Fitzpatrick TB, Lew RA, Kopf AW, Mihm MC (1983). Factors associated with death from melanoma from 2 to 5 years following diagnosis in clinical stage I patients. J Invest Dermatol.

[6] Atkins MB, Lotze MT, Dutcher JP, Fisher RI, Weiss G, Margolin K (1999). High-dose recombinant interleukin 2 therapy for patients with metastatic melanoma: analysis of 270 patients treated between 1985 and 1993. J Clin Oncol.

[7] Harlin H, Kuna TV, Peterson AC, Meng Y, Gajewski TF (2006). Tumor progression despite massive influx of activated CD8(+) T cells in a patient with malignant melanoma ascites. Cancer Immunol Immunother.

[8] Spranger S, Spaapen RM, Zha Y, Williams J, Meng Y, Ha TT (2013). Up-regulation of PD-L1, IDO, and T(regs) in the melanoma tumor microenvironment is driven by CD8(+) T cells. Sci Transl Med.

[9] Strome SE, Dong H, Tamura H, Voss SG, Flies DB, Tamada K (2003). B7-H1 blockade augments adoptive T-cell immunotherapy for squamous cell carcinoma. Cancer Res.

[10] Wong RM, Scotland RR, Lau RL, Wang C, Korman AJ, Kast WM (2007). Programmed death-1 blockade enhances expansion and functional capacity of human melanoma antigen-specific CTLs. Int Immunol.

[11] Yao S, Zhu Y, Chen L (2013). Advances in targeting cell surface signalling molecules for immune modulation. Nat Rev Drug Discov.

[12] Wing K, Onishi Y, Prieto-Martin P, Yamaguchi T, Miyara M, Fehervari Z (2008). CTLA-4 control over Foxp3+ regulatory T cell function. Science.

[13] Peggs KS, Quezada SA, Chambers CA, Korman AJ, Allison JP (2009). Blockade of CTLA-4 on both effector and regulatory T cell compartments contributes to the antitumor activity of anti-CTLA-4 antibodies. J Exp Med.

[14] Phan GQ, Yang JC, Sherry RM, Hwu P, Topalian SL, Schwartzentruber DJ (2003). Cancer regression and autoimmunity induced by cytotoxic T lymphocyte-associated antigen 4 blockade in patients with metastatic melanoma. Proc Natl Acad Sci U S A.

[15] Attia P, Phan GQ, Maker AV, Robinson MR, Quezado MM, Yang JC (2005). Autoimmunity correlates with tumor regression in patients with metastatic melanoma treated with anti-cytotoxic T-lymphocyte antigen-4. J Clin Oncol.

[16] Maker AV, Yang JC, Sherry RM, Topalian SL, Kammula US, Royal RE (2006). Intrapatient dose escalation of anti-CTLA-4 antibody in patients with metastatic melanoma. J Immunother.

[17] Hodi FS, O'Day SJ, McDermott DF, Weber RW, Sosman JA, Haanen JB (2010). Improved survival with ipilimumab in patients with metastatic melanoma. N Engl J Med.

[18] Robert C, Thomas L, Bondarenko I, O'Day S, Weber J, Garbe C (2011). Ipilimumab plus dacarbazine for previously untreated metastatic melanoma. N Engl J Med.

[19] Schadendorf D, Hodi FS, Robert C, Weber JS, Margolin K, Hamid O (2015). Pooled analysis of long-term survival data from phase II and phase III trials of ipilimumab in unresectable or metastatic melanoma. J Clin Oncol.

[20] Camacho LH, Antonia S, Sosman J, Kirkwood JM, Gajewski TF, Redman B (2009). Phase I/II trial of tremelimumab in patients with metastatic melanoma. J Clin Oncol.

[21] Ribas A, Kefford R, Marshall MA, Punt CJ, Haanen JB, Marmol M (2013). Phase III randomized clinical trial comparing tremelimumab with standard-of-care chemotherapy in patients with advanced melanoma. J Clin Oncol.

[22] Bertrand A, Kostine M, Barnetche T, Truchetet ME, Schaeverbeke T (2015). Immune related adverse events associated with anti-CTLA-4 antibodies: systematic review and meta-analysis. BMC Med.

[23] Weber JS, Kahler KC, Hauschild A (2012). Management of immune-related adverse events and kinetics of response with ipilimumab. J Clin Oncol.

[24] Wolchok JD, Hoos A, O'Day S, Weber JS, Hamid O, Lebbe C (2009). Guidelines for the evaluation of immune therapy activity in solid tumors: immune-related response criteria. Clin Cancer Res.

[25] Brahmer JR, Drake CG, Wollner I, Powderly JD, Picus J, Sharfman WH (2010). Phase I study of single-agent anti-programmed death-1 (MDX-1106) in refractory solid tumors: safety, clinical activity, pharmacodynamics, and immunologic correlates. J Clin Oncol.

[26] Topalian SL, Hodi FS, Brahmer JR, Gettinger SN, Smith DC, McDermott DF (2012). Safety, activity, and immune correlates of anti-PD-1 antibody in cancer. N Engl J Med.

[27] Topalian SL, Sznol M, McDermott DF, Kluger HM, Carvajal RD, Sharfman WH (2014). Survival, durable tumor remission, and long-term safety in patients with advanced melanoma receiving nivolumab. J Clin Oncol.

[28] Hodi FS, Sznol M, Kluger HM, McDermott DF, Carvajal RD, Lawrence DP (2014). Long-term survival of ipilimumab-naive patients (pts) with advanced melanoma (MEL) treated with nivolumab (anti-PD-1, BMS-936558, ONO-4538) in a phase I trial. J Clin Oncol.

[29] Hamid O, Robert C, Daud A, Hodi FS, Hwu WJ, Kefford R (2013). Safety and tumor responses with lambrolizumab (anti-PD-1) in melanoma. N Engl J Med.

[30] Daud A, Ribas A, Robert C, Hodi FS, Wolchok JD, Joshua AM, et al. Long-term efficacy of pembrolizumab (pembro; MK-3475) in a pooled analysis of 655 patients (pts) with advanced melanoma (MEL) enrolled in KEYNOTE-001. J Clin Oncol. 2015;33:(suppl; abstr 9005).

[31] Weber JS, D'Angelo SP, Minor D, Hodi FS, Gutzmer R, Neyns B (2015). Nivolumab versus chemotherapy in patients with advanced melanoma who progressed after anti-CTLA-4 treatment (CheckMate 037): a randomised, controlled, open-label, phase 3 trial. Lancet Oncol.

[32] Ribas A, Puzanov I, Dummer R, Schadendorf D, Hamid O, Robert C (2015). Pembrolizumab versus investigator-choice chemotherapy for ipilimumab-refractory melanoma (KEYNOTE-002): a randomised, controlled, phase 2 trial. Lancet Oncol.

[33] Robert C, Ribas A, Wolchok JD, Hodi FS, Hamid O, Kefford R (2014). Anti-programmed-death-receptor-1 treatment with pembrolizumab in ipilimumab-refractory advanced melanoma: a randomised dose-comparison cohort of a phase 1 trial. Lancet.

[34] Lutzky J, Antonia SJ, Blake-Haskins A, Li X, Robbins PB, Shalabi AM, et al. A phase 1 study of MEDI4736, an anti-PD-L1 antibody, in patients with advanced solid tumors. J Clin Oncol. 2014;32(5s):(suppl; abstr 3001^).

[35] Hamid O, Sosman JA, Lawrence DP, Sullivan RJ, Ibrahim N, Kluger HM, et al. Clinical activity, safety, and biomarkers of MPDL3280A, an engineered PD-L1 antibody in patients with locally advanced or metastatic melanoma (mM). J Clin Oncol. 2013;31:(suppl; abstr 9010).

[36] Robert C, Schachter J, Long GV, Arance A, Grob JJ, Mortier L (2015). Pembrolizumab versus ipilimumab in advanced melanoma. N Engl J Med.

[37] Larkin J, Chiarion-Sileni V, Gonzalez R, Grob JJ, Cowey CL, Lao CD (2015). Combined nivolumab and ipilimumab or monotherapy in untreated melanoma. N Engl J Med.

[38] Gibney GT, Atkins MB (2015). Swinging for the fences: long-term survival with ipilimumab in metastatic melanoma. J Clin Oncol.

[39] Margolin K, Ernstoff MS, Hamid O, Lawrence D, McDermott D, Puzanov I (2012). Ipilimumab in patients with melanoma and brain metastases: an open-label, phase 2 trial. Lancet Oncol.

[40] Kluger HM, Zito CR, Barr ML, Baine MK, Chiang VL, Sznol M (2015). Characterization of PD-L1 expression and associated T-cell infiltrates in metastatic melanoma samples from variable anatomic sites. Clin Cancer Res.

[41] Kluger HM, Goldberg SB, Sznol M, Tsiouris J, Vortmeyer A, Jilaveanu L, et al. Safety and activity of pembrolizumab in melanoma patients with untreated brain metastases. J Clin Oncol. 2015;33:(suppl; abstr 9009).

[42] Balch CM, Gershenwald JE, Soong SJ, Thompson JF, Atkins MB, Byrd DR (2009). Final version of 2009 AJCC melanoma staging and classification. J Clin Oncol.

[43] Kirkwood JM, Manola J, Ibrahim J, Sondak V, Ernstoff MS, Rao U (2004). A pooled analysis of eastern cooperative oncology group and intergroup trials of adjuvant high-dose interferon for melanoma. Clin Cancer Res.

[44] Eggermont AM, Suciu S, Testori A, Santinami M, Kruit WH, Marsden J (2012). Long-term results of the randomized phase III trial EORTC 18991 of adjuvant therapy with pegylated interferon alfa-2b versus observation in resected stage III melanoma. J Clin Oncol.

[45] Tarhini AA, Thalanayar PM (2014). Melanoma adjuvant therapy. Hematol Oncol Clin North Am.

[46] Flaherty LE, Othus M, Atkins MB, Tuthill RJ, Thompson JA, Vetto JT (2014). Southwest Oncology Group S0008: a phase III trial of high-dose interferon Alfa-2b versus cisplatin, vinblastine, and dacarbazine, plus interleukin-2 and interferon in patients with high-risk melanoma--an intergroup study of cancer and leukemia Group B, Children's Oncology Group, Eastern Cooperative Oncology Group, and Southwest Oncology Group. J Clin Oncol.

[47] Eggermont AM, Chiarion-Sileni V, Grob JJ, Dummer R, Wolchok JD, Schmidt H (2015). Adjuvant ipilimumab versus placebo after complete resection of high-risk stage III melanoma (EORTC 18071): a randomised, double-blind, phase 3 trial. Lancet Oncol.

[48] Tarhini AA (2014). Adjuvant Therapy for High-Risk Melanoma. Am J Hematol Oncol.

[49] Gibney GT, Kudchadkar RR, DeConti RC, Thebeau MS, Czupryn MP, Tetteh L (2015). Safety, correlative markers, and clinical results of adjuvant nivolumab in combination with vaccine in resected high-risk metastatic melanoma. Clin Cancer Res.

[50] Curran MA, Montalvo W, Yagita H, Allison JP (2010). PD-1 and CTLA-4 combination blockade expands infiltrating T cells and reduces regulatory T and myeloid cells within B16 melanoma tumors. Proc Natl Acad Sci U S A.

[51] Wolchok JD, Kluger H, Callahan MK, Postow MA, Rizvi NA, Lesokhin AM (2013). Nivolumab plus ipilimumab in advanced melanoma. N Engl J Med.

[52] Sznol M, Kluger HM, Callahan MK, Postow MA, Gordon RA, Segal NH, et al. Survival, response duration, and activity by BRAF mutation (MT) status of nivolumab (NIVO, anti-PD-1, BMS-936558, ONO-4538) and ipilimumab (IPI) concurrent therapy in advanced melanoma (MEL). J Clin Oncol. 2014;32(5s):(suppl; abstr LBA9003^).

[53] Postow MA, Chesney J, Pavlick AC, Robert C, Grossmann K, McDermott D (2015). Nivolumab and ipilimumab versus ipilimumab in untreated melanoma. N Engl J Med.

[54] Hodi FS, Gibney GT, Sullivan R, Sosman JA, Slingluff CL, Lawrence DP, et al. An open-label, randomized, phase 2 study of nivolumab given sequentially with ipilimumab in patients with advanced melanoma (Checkmate 064). ECCO-ESMO. 25–29 September 2015. 2015:abstract 23LBA.

[55] Atkins MB, Choueiri TK, Hodi FS, Thompson JA, Hwu W, McDermott DF, et al. Pembrolizumab (MK-3475) plus low-dose ipilimumab (IPI) in patients (pts) with advanced melanoma (MEL) or renal cell carcinoma (RCC): Data from the KEYNOTE-029 phase 1 study. J Clin Oncol. 2015;33(5s):(suppl; abstr 3009).

[56] Kudchadkar RR, Gibney GT, Dorman D, Merek S, Ramadan H, Chen A, et al. A phase IB study of ipilimumab with peginterferon alfa-2b for patients with unresectable stages IIIB/C/IV melanoma. J Clin Oncol. 2014;32(5s):(suppl; abstr 9098).

[57] Hodi FS, Lee S, McDermott DF, Rao UN, Butterfield LH, Tarhini AA (2014). Ipilimumab plus sargramostim vs ipilimumab alone for treatment of metastatic melanoma: a randomized clinical trial. JAMA.

[58] Zarour HM, Tawbi H, Tarhini AA, Wang H, Sander C, Rose A, et al. Study of anti-PD-1 antibody pembrolizumab and pegylated-interferon alfa-2b (Peg-IFN) for advanced melanoma. J Clin Oncol. 2015;33:(suppl; abstr e20018).

[59] Andtbacka RH, Kaufman HL, Collichio F, Amatruda T, Senzer N, Chesney J (2015). Talimogene laherparepvec improves durable response rate in patients with advanced melanoma. J Clin Oncol.

[60] Puzanov I, Milhem MM, Andtbacka RHI, Minor DR, Hamid O, Li A, et al. Survival, safety, and response patterns in a phase 1b multicenter trial of talimogene laherparepvec (T-VEC) and ipilimumab (IPI) in previously untreated, unresected stage IIIB-IV melanoma. J Clin Oncol. 2015;33:(suppl; abstr 9063).

[61] Uyttenhove C, Pilotte L, Theate I, Stroobant V, Colau D, Parmentier N (2003). Evidence for a tumoral immune resistance mechanism based on tryptophan degradation by indoleamine 2,3-dioxygenase. Nat Med.

[62] Liu X, Shin N, Koblish HK, Yang G, Wang Q, Wang K (2010). Selective inhibition of IDO1 effectively regulates mediators of antitumor immunity. Blood.

[63] Beatty GL DP, Clark J, Shi JG, Newton RC, Schaub R, Maleski J, et al. Phase I study of the safety, pharmacokinetics (PK), and pharmacodynamics (PD) of the oral inhibitor of indoleamine 2,3-dioxygenase (IDO1) INCB024360 in patients (pts) with advanced malignancies. J Clin Oncol 31, 2013 (suppl; abstr 3025). http://meetinglibrary.asco.org/content/115661-132. Accessed 21 Nov 2015.

[64] Gibney GT, Hamid O, Lutzky J, Olszanski AJ, Gangadhar TC, Gajewski TF, et al. Updated results from a phase 1/2 study of epacadostat (INCB024360) in combination with ipilimumab in patients with metastatic melanoma. ECCO-ESMO 2015: abstract 511. 2015. http://www.europeancancercongress.org/Scientific-Programme/Abstract-search?abstractid=23278. Accessed 22 Jan 2016.

[65] Spranger S, Koblish HK, Horton B, Scherle PA, Newton R, Gajewski TF (2014). Mechanism of tumor rejection with doublets of CTLA-4, PD-1/PD-L1, or IDO blockade involves restored IL-2 production and proliferation of CD8(+) T cells directly within the tumor microenvironment. J Immunother Cancer.

[66] Gangadhar TC, Hamid O, Smith DC, Bauer TM, Wasser JS, Luke JJ (2015). Preliminary results from a Phase I/II study of epacadostat (incb024360) in combination with pembrolizumab in patients with selected advanced cancers. J Immunother Cancer.

[67] Woo SR, Turnis ME, Goldberg MV, Bankoti J, Selby M, Nirschl CJ (2012). Immune inhibitory molecules LAG-3 and PD-1 synergistically regulate T-cell function to promote tumoral immune escape. Cancer Res.

[68] Okazaki T, Okazaki IM, Wang J, Sugiura D, Nakaki F, Yoshida T (2011). PD-1 and LAG-3 inhibitory co-receptors act synergistically to prevent autoimmunity in mice. J Exp Med.

[69] Taube JM, Young GD, McMiller TL, Chen S, Salas JT, Pritchard TS (2015). Differential expression of immune-regulatory genes associated with PD-L1 display in melanoma: implications for PD-1 pathway blockade. Clin Cancer Res.

[70] Sabatino M, Kim-Schulze S, Panelli MC, Stroncek D, Wang E, Taback B (2009). Serum vascular endothelial growth factor and fibronectin predict clinical response to high-dose interleukin-2 therapy. J Clin Oncol.

[71] Panelli MC, Wang E, Phan G, Puhlmann M, Miller L, Ohnmacht GA, et al. Gene-expression profiling of the response of peripheral blood mononuclear cells and melanoma metastases to systemic IL-2 administration. Genome Biol. 2002;3(7):RESEARCH0035. 10.1186/gb-2002-3-7-research0035 PMC126240 12184809

[72] Mahoney KM, Atkins MB (2014). Prognostic and predictive markers for the new immunotherapies. Oncology (Williston Park).

[73] Patel SP, Kurzrock R (2015). PD-L1 expression as a predictive biomarker in cancer immunotherapy. Mol Cancer Ther.

[74] Garon EB, Rizvi NA, Hui R, Leighl N, Balmanoukian AS, Eder JP (2015). Pembrolizumab for the treatment of non-small-cell lung cancer. N Engl J Med.

[75] Borghaei H, Paz-Ares L, Horn L, Spigel DR, Steins M, Ready NE (2015). Nivolumab versus docetaxel in advanced nonsquamous non-small-cell lung cancer. N Engl J Med.

[76] Tumeh PC, Harview CL, Yearley JH, Shintaku IP, Taylor EJ, Robert L (2014). PD-1 blockade induces responses by inhibiting adaptive immune resistance. Nature.

[77] Taube JM, Anders RA, Young GD, Xu H, Sharma R, McMiller TL, et al. Colocalization of inflammatory response with B7-h1 expression in human melanocytic lesions supports an adaptive resistance mechanism of immune escape. Sci Transl Med. 2012;4:127ra137. 10.1126/scitranslmed.3003689 PMC3568523 22461641

[78] Taube JM, Klein A, Brahmer JR, Xu H, Pan X, Kim JH (2014). Association of PD-1, PD-1 ligands, and other features of the tumor immune microenvironment with response to anti-PD-1 therapy. Clin Cancer Res.

[79] Ribas A, Robert C, Hodi FS, Wolchok JD, Joshua AM, Hwu W-J, et al. Association of response to programmed death receptor 1 (PD-1) blockade with pembrolizumab (MK-3475) with an interferon-inflammatory immune gene signature. J Clin Oncol. 2015;33:(suppl; abstr 3001).

[80] Snyder A, Makarov V, Merghoub T, Yuan J, Zaretsky JM, Desrichard A (2014). Genetic basis for clinical response to CTLA-4 blockade in melanoma. N Engl J Med.

[81] Van Allen EM, Miao D, Schilling B, Shukla SA, Blank C, Zimmer L (2015). Genomic correlates of response to CTLA-4 blockade in metastatic melanoma. Science.

[82] Rooney MS, Shukla SA, Wu CJ, Getz G, Hacohen N (2015). Molecular and genetic properties of tumors associated with local immune cytolytic activity. Cell.

[83] Rizvi NA, Hellmann MD, Snyder A, Kvistborg P, Makarov V, Havel JJ (2015). Cancer immunology. Mutational landscape determines sensitivity to PD-1 blockade in non-small cell lung cancer. Science.

[84] F Freeman-Keller ML, Weber JS. Nivolumab in resected and unresectable melanoma: Immune-related adverse events and association with survival outcomes. J Clin Oncol. 2015;33:(suppl; abstr 9028). 10.1158/1078-0432.CCR-15-1136 PMC4755809 26446948

[85] Horvat TZ, Adel NG, Dang TO, Momtaz P, Postow MA, Callahan MK (2015). Immune-related adverse events, need for systemic immunosuppression, and effects on survival and time to treatment failure in patients with melanoma treated with ipilimumab at Memorial Sloan Kettering Cancer Center. J Clin Oncol.

